# Men’s experiences of multiple long-term conditions and/or disability in the UK Game of Stones weight management trial: a mixed-methods evaluation

**DOI:** 10.1136/bmjopen-2025-113802

**Published:** 2026-09-15

**Authors:** Lisa Macaulay, Catriona O’Dolan, James Swingler, Claire Torrens, Alice MacLean, Katrina Turner, Alison Avenell, Seonaidh Cotton, Stephan U Dombrowski, Cindy Gray, Kate Hunt, Frank Kee, Michelle C McKinley, Graeme MacLennan, Pat Hoddinott

**Affiliations:** 1 Faculty of Health Sciences and Sport, University of Stirling, Stirling, UK; 2 Research Centre for Health, Glasgow Caledonian University, Glasgow, UK; 3 Aberdeen Centre for Evaluation, University of Aberdeen, Aberdeen, UK; 4 Institute for Social Marketing and Health, University of Stirling, Stirling, UK; 5 Centre for Academic Primary Care, University of Bristol, Bristol, UK; 6 Centre for Healthcare Randomised Trials, University of Aberdeen, Aberdeen, UK; 7 Faculty of Kinesiology, University of New Brunswick, Fredericton, New Brunswick, Canada; 8 School of Social and Political Sciences, University of Glasgow, Glasgow, Strathclyde, UK; 9 Centre for Public Health, Queen’s University Belfast, Belfast, UK; 10 Queen’s University Belfast, Belfast, UK

**Keywords:** Obesity, PUBLIC HEALTH, QUALITATIVE RESEARCH, PREVENTIVE MEDICINE, Overweight, Multimorbidity

## Abstract

**Objectives:**

To explore experiences, health outcomes and retention of men with multiple long-term conditions (MLTCs) and/or disability within the Game of Stones weight management randomised controlled trial (RCT).

**Design:**

Mixed-methods process evaluation within an RCT where secondary outcomes included the Weight Self-Stigma Questionnaire, EuroQol 5-Dimension 5-Level (EQ-5D-5L), EQ-5D-5L anxiety and depression subscale, Patient Health Questionnaire-4 and retention. Semistructured interviews were conducted at 12 months and analysed using the framework method.

**Setting:**

Conducted across three UK trial centres: Belfast, Bristol and Glasgow.

**Participants:**

585 men with obesity (mean (SD) age, 50.7 (13.3) years) were randomised to one of three groups: behavioural text messages with financial incentives, texts alone or waiting-list control. Interviews were conducted with 54 participants from the two intervention groups.

**Results:**

235 (40%) participants lived with MLTCs, 181 (31%) had a single condition, 167 (29%) had no conditions and 165 (29%) had a disability. Of those with MLTCs, 99 were disabled and 93 were living in deprived areas. Participants with MLTCs and/or disability were older, fewer had a degree-level qualification and fewer were in full-time work. Retention at 12 months was higher for men with disability (76%) or no long-term conditions (75%) and lower for men with diabetes (65%). Self-reported weight stigma, well-being and quality-of-life scores improved or stayed the same for men living with MLTCs in the intervention groups; however, results for anxiety and depression screening scores were inconsistent. Participant experiences indicated complex dynamic health, social and life situations which could provide motivation to lose weight for some but not others. Hospitalisation and poor mobility, with inability to exercise, were demotivating for making changes to reach weight loss targets.

**Conclusions:**

Men living with MLTCs and/or disability varied from very successful weight loss and improved health to not prioritising or feeling helped by the programme or disengagement due to immobility or diabetes.

**Trial registration number:**

isrctn.org Identifier: ISRCTN91974895.

**STRENGTHS AND LIMITATIONS OF THIS STUDY**

- This study provides insights into the experiences of men living with multiple long-term conditions (MLTC) and/or disability, who are under-represented in behavioural weight management trials.
- The length of the questionnaire, designed to reduce participant burden and guided by public and patient involvement, prevented collection of data-related to all obesity-related conditions.
- The prevalence of MLTCs was higher at the end of the trial than at the start because several men reported new diagnoses during the trial, for example, hypertension.
- Although it has been shown that obesity increases the risk of dementia, this condition was under-represented in Game of Stones given that cognitive ability to understand text messages was an inclusion criterion.

## Introduction

 Obesity increases the risk of type 2 diabetes, heart disease, stroke, mobility problems and some cancers, leading to multimorbidity, and its prevalence is rising.^1^ An observational multicohort study examined obesity as a shared risk factor for common diseases, associations between obesity-related diseases and the role of obesity in the development of complex multimorbidity (defined as four or more comorbid diseases).^2^ The study showed that obesity was associated with 21 major diseases, with the degree of obesity associated in a dose–response relationship. The authors concluded that obesity is associated with diverse disease burdens, and targeting obesity in multimorbidity prevention may be more effective than targeting the diseases on an individual disease basis. Treating chronic diseases without addressing the prevention and management of obesity can promote multimorbidity, as causal relationships are complex and multidirectional.^3^

Sustainable weight loss interventions with broad reach are needed for men who do not like or cannot access weight loss groups or services. Men engage less often than women in weight loss interventions,^4^ yet men die sooner than women and year-on-year mortality improvements have slowed, particularly for those living in disadvantaged areas.^5 6^ UK Office for National Statistics data show that, since the COVID-19 pandemic, overall life expectancy has decreased more for men than women, returning to below the 2010–2012 level, and is falling in all four UK nations for 2020–2022 compared with 2017–2019.^5^ The proportion of life spent in poor health has increased over time for both sexes. Both life expectancy and the proportion of life lived in good health are closely linked to deprivation: the greater the deprivation, the worse the outcomes.^7^

The number of people living with multiple long-term conditions (MLTCs) is rising globally, posing challenges for patients and for the complexity and volume of healthcare work and for health and social care research.^2 8–10^ In the UK, there has been a 5% rise in the number of people reporting a disability in the last decade, from 19% in 2012/2013 to 24% in 2022/2023, which generates socioeconomic disparities.^11^ There is an evidence gap in existing systematic reviews for men living with MLTCs, disability and obesity who participate in weight management intervention trials.^12 13^ A systematic review of digital interventions for patients with multimorbidity in 2020 did not include weight management trials.^13^ Many trials of behavioural weight management interventions focus on participants with single non-communicable conditions.^14 15^ However, a large UK primary care cohort study highlighted the high prevalence of comorbidities in patients with type 2 diabetes and the consequences of metabolic syndrome where disease risk factors cluster.^16^ A qualitative systematic review found self-management support interventions (but not obesity specific) for men with long-term conditions to be acceptable.^17^ There is therefore a knowledge gap about both the impact of participating in weight management trials and the lived experience for men who are living with obesity and MLTCs.

The Game of Stones weight management trial (hereafter referred to as the trial) was designed to appeal to any man living with obesity, but in particular men who face challenges attending intense in-person weight management programmes or prefer not to.^18^ The trial randomised 585 men (mean (SD) age, 50.7 (13.3) years) with obesity to receive (1) daily behavioural text messages with endowment financial incentives where money was lost for not meeting three 5%–10% weight loss targets over 12 months, (2) text messages alone or (3) a waiting list control. The text message design incorporated behaviour change strategies to predominantly support motivation, self-regulation and long-term maintenance of behaviour change using a person-centred approach. The results of the trial showed that compared with the control group, men in the text messages with financial incentives group had a significantly larger mean per cent weight loss after 12 months (mean difference −3.2% (97.5% CI −4.6% to −1.9%, p<0.001)); the text messages alone did not significantly affect per cent weight loss (mean difference −1.4% (97.5% CI −2.9% to 0.0%, p=0.053)). Examples of the text messages and a full description of the trial findings are presented elsewhere.^19^ The trial population was unusual for behavioural weight management trials in men,^20^ with 227 (39%) living in more disadvantaged areas and 416 (71%) with at least one obesity-related comorbidity. The trial recruited and assessed participants from July 2021 to May 2023 which spanned the COVID-19 pandemic.

Participants reported 366 adverse events during the trial, classified using the MedDRA (v26.1) (https://www.meddra.org/) classification, with 23 (6.3%) classified as serious.^19^ None were considered associated with participation in the trial. The most common categories of adverse events were 83 (23%) infections, 58 (17%) social harms (such as bereavement) and 39 (11%) musculoskeletal and connective tissue conditions. Prespecified subgroup analyses for moderators of the primary outcome of per cent weight change at 12 months (socioeconomic factors and health and well-being status, including multiple or single long-term conditions, disability, mental health condition, diabetes and quality of life) showed no difference for either intervention group compared with the control group.^21^ Game of Stones was shown to be equally effective across a variety of different subpopulations, without exacerbating socioeconomic inequalities.

The focus of this paper is the exploratory mixed-methods trial process evaluation findings. The aims are (1) to explore trial experiences, health outcomes and retention for men living with MLTCs and/or disability within the trial after 12 months and (2) to understand how living with both obesity and MLTCs and/or disability impacted on the lived experiences for men in the trial intervention groups.

## Methods

A mixed-methods exploratory process evaluation was included in the trial protocol,^22^ which triangulated and integrated trial data sources, looking for agreement, disconfirming perspectives and possible explanations that might help to understand the results.^19 23 24^ Key methods and statistical analysis of secondary outcomes relevant to this paper are summarised below and in the trial protocol paper^22^ with availability of the protocol in online supplemental file S1: Game of Stones Protocol. The trial recruited 585 men aged over 18 years with a body mass index equal to or greater than 30 kg/m^2^ between 12 July 2021 and 30 May 2022, in and around three UK cities, purposively selected to include areas of socioeconomic disadvantage. All participants provided written informed consent. There were two recruitment strategies: (1) community outreach (posters, leaflets, information stands) and (2) GP opt-in referral letters. All participants were assessed at baseline and 12 months; participants in the text messages with incentives and text messages alone groups were additionally followed up at 3 and 6 months for weight measurements, unexpected harms and benefits.

To establish participants’ long-term condition and disability status at baseline, men were asked in the participant questionnaire to confirm whether a doctor had ever told them that they have any of the following obesity-related conditions by ticking a box: stroke/ministroke; high blood pressure; heart condition, for example, angina or atrial fibrillation; diabetes; cancer; arthritis; or a mental health condition. Living with MLTCs was defined as answering ‘yes’ to two or more of these conditions. Disability was defined according to the UK Office for National Statistics standard^25^ with a ‘yes’ required to both of the following questions:

1. Do you have any physical or mental health conditions or illnesses lasting or expected to last 12 months or more?
2. Do any of your conditions or illnesses reduce your ability to carry-out day-to-day activities?

UK country-specific measures for the Index of Multiple Deprivations were used, which describe participant postcode area of residence according to five quintiles.^19^

The exploratory process evaluation findings relating specifically to participants with a mental health condition (n=146, 25%) are reported elsewhere but concluded that mental health difficulties were experienced in different ways by men during the trial, were unique to the individual and were a barrier to behaviour change for some but not for others.^26^

At the 3-month, 6-month and 12-month weight assessments, men were asked ‘Has anything unexpected happened since your last appointment as a result of taking part in the study (these can be either helpful or harmful consequences)?’ The researcher recorded a ‘no’ or ‘yes’ depending on their response and if yes, detailed the response on the case report form. Adverse events were classified according to a protocol and are reported descriptively with the trial results.^19^ These data are referred to as ‘Harms and Benefits data’ in accordance with Consolidated Standards of Reporting Trials Guidance^27^ and guidance for weight management programmes.^28^

For the analysis of quantitative data, the baseline characteristics by trial group, general practice or community recruitment route, long-term conditions and mental health status are reported elsewhere.^19 29^ 583 participants completed the baseline questionnaire. The two participants who did not return a completed baseline questionnaire were excluded from the results of this paper. Baseline participant characteristics are described for the whole cohort, and categorised as MLTCs, disability and no long-term conditions or disability. Participants were categorised into one of three levels (none, one or multiple) according to self-report (by questionnaire) of long-term conditions at baseline. For participants with MLTCs, the participant accounts of harms and benefits documented by researchers at weight assessments were merged with trial data for baseline characteristics and outcome data. All quantitative results are presented as frequencies and percentages for categorical, and as the mean, SD and count for continuous variables, respectively. All findings are to be interpreted cautiously and with the knowledge that no formal statistical analyses were undertaken.

Qualitative interviews were conducted after 12-month primary outcome data collection (July 2022–May 2023). Men were purposively selected to ensure a diverse sample representative of each trial centre, intervention group, differing deprivation status in addition to those self-reporting long-term conditions. A topic guide of questions and prompts was used, for example: Has anything made it difficult for you to take part in Game of Stones? and What did you think of being offered money for reaching weight loss targets? (online supplemental file S2: Game of Stones 12 Month Interview Topic Guide). Interviews were conducted remotely (video or telephone) by three researchers with qualitative expertise (CT, CO’D and KM) with men in the intervention groups they had not met during the trial. Participant interviews were transcribed and uploaded into QSR NVivo (v20). Three researchers (CT, CO’D and AM) independently developed a coding frame (agreed through team discussion) and identified key themes after reading a diverse sample of interviews. Analysis was undertaken by a core team (CO’D, CT, AM and KT) who explored and refined a hypothesised theory of change, guided by the five stages of the framework approach^30^: familiarisation; identifying a thematic framework; indexing; charting; mapping and interpretation. Attributes for trial participants were assigned for sociodemographic data (eg, Index of Multiple Deprivations); health and well-being (MLTCs; mental health conditions) and linked to interview transcripts. After data lock, weight loss outcome data were also assigned as an attribute (ie, met some/all targets or met no targets) and linked to the transcripts. Matrices were constructed in NVivo and the assigned attributes added to identify patterns with the aim of contextualising and interpreting participant outcomes. The qualitative analysis was completed after the absence of any differential effectiveness for subgroup moderator analyses, according to the presence/absence of one or more long-term conditions was known.^31^ Triangulation with harms and benefits data and researcher fieldnotes was undertaken as the final step by LM, PH and KT, to search for alternative or disconfirming perspectives. The harms and benefits spreadsheets linked to participant attributes were read and re-read to identify themes and patterns in the data. The multidisciplinary trial team contributed their expertise to data interpretation. In the results, participant quotations illustrate how MLTCs impacted men’s weight loss during the trial. Interview data are reported ‘verbatim’ with labels for trial group and whether weight loss targets were met: ≥5% loss from baseline at 3 months; ≥10% loss at 6 months and ≥10% loss maintained at 12 months. Where appropriate, fieldworker notes detailing participants’ responses for the harms and benefits question have been integrated with the interview data.

### Patient and public involvement

Patient and public involvement (PPI) in Game of Stones began in 2011 with the involvement of Men’s Health Forum Charities with the ROMEO study evidence synthesis of weight loss interventions for men with obesity,^4^ which informed the Game of Stones feasibility study. Continuous and responsive public involvement continued through the feasibility study (2017–2018) with 121 contributors involved in activities including: naming the study, development of the text message intervention and trial materials, and attending study oversight groups. PPI involvement in the full trial has continued throughout. PPI members attended the project management group and trial steering group which met 16 and 7 times, respectively. Their input was invaluable in refining the trial protocol, reviewing trial documents, maximising recruitment, monitoring trial progress, advising on protocol adaptations, coauthoring outputs and advising on dissemination.

## Results

Baseline characteristics for participants with MLTCs (including those with a mental health condition) and meeting the UK Office for National Statistics definition of disability^25^ were compared with men who reported no long-term conditions and with no disability, respectively (table 1, with the full data set in online supplemental file S3: Baseline characteristics according to MLTC and disability status). Notable differences between men in the three categories of interest were for age, education, employment, relationship, household and comorbidities. Men with MLTCs and/or with a disability were older, fewer had a degree level qualification, more were retired and fewer were in full-time work. A higher proportion of men with a disability reported living alone or their relationship status as single. A higher proportion of men with MLTCs reported having high blood pressure, heart conditions and diabetes compared with men with a disability.

**Table 1:** Baseline characteristics for men living with multiple long-term conditions, disability or with no long-term conditions

	Multiple long-term conditions (n=235)	Disability† (n=165)	No long-term conditions or disability (n=152)	Trial participants‡ (n=583)
Age*, mean (SD); n	58.4 (11.4); 235	52.9 (13.5); 164	43.5 (11.0); 152	50.7 (13.3); 582
Index of Multiple Deprivation Category, n (%)	n=234	n=164	n=150	n=579
Most deprived	59 (25.2)	47 (28.7)	34 (22.7)	133 (23)
More deprived	34 (14.5)	24 (14.6)	23 (15.3)	92 (16)
Deprived	38 (16.2)	32 (19.5)	16 (10.7)	87 (15)
Less deprived	35 (15.0)	27 (16.5)	35 (23.3)	110 (19)
Least deprived	68 (29.1)	34 (20.7)	42 (28.0)	157 (27)
Relationship status*, n (%)	n=231	n=163	n=152	n=574
Single (never married; never in a civil partnership)	25 (10.8)	32 (19.6)	22 (14.5)	76 (13)
Comorbidities*, n (%)				n=583
High blood pressure	200 (85.1)	97 (58.8)	–	262 (45)
Mental health condition	82 (34.9)	71 (43.0)	–	146 (25)
Arthritis	115 (48.9)	70 (42.4)	–	142 (24)
Diabetes	90 (38.3)	40 (24.2)	–	104 (18)
Heart condition such as angina or atrial fibrillation	82 (34.9)	29 (17.6)	–	91 (16)
Stroke (including transient ischaemic attack)	19 (8.1)	8 (4.8)	–	20 (3.4)
Cancer	15 (6.4)	6 (3.6)	–	19 (3.2)
Household composition*, n (%)				n=583
Lives alone	32 (13.6)	30 (18.2)	13 (8.6)	68 (12)
Highest educational qualification*, n (%)	n=196	n=145	n=143	n=522
Degree level or above	79 (40.3)	59 (40.7)	82 (57.3)	249 (48)
Another kind of qualification	117 (59.7)	86 (59.3)	61 (42.7)	273 (52)
Employment status*, n (%)	n=227	n=160	n=148	n=568
Paid job, full time (30+ hours per week)	95 (41.9)	75 (46.9)	115 (76.2)	334 (59)
Retired	73 (32.2)	32 (20.0)	7 (4.6)	98 (17)
Not in paid work due to illness or disability	18 (7.9)	27 (16.9)	–	29 (5.1)

* Self-report data. Some were missing for Index of Multiple Deprivation, data for some postcodes were not available.

† Meets Office for National Statistics definition of disability.

‡ Two trial participants did not complete baseline questionnaires.

**Figure 1:**
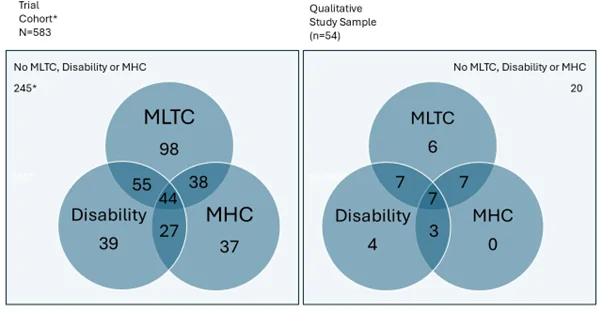
Overlap of multiple long-term conditions (MLTCs), mental health conditions (MHCs) and disability among trial and qualitative interview participants. *Excludes two participants who could not be classified at baseline due to missing information.

The overlap between men with MLTCs, men with disability and men reporting that they had ever had a doctor-diagnosed mental health condition is summarised in figure 1 for the trial population and for the qualitative interview sample. Table 2 presents the overlap between living with multiple, single and no long-term conditions with disability and for living in the two more disadvantaged quintiles for the Index of Multiple Deprivation. Of the 235 men living with MLTCs, 99 (42%) met the UK Office for National Statistics criteria for disability, a further 34 (14%) responded yes to the first disability question and 93 (40%) were living in the two more deprived postcode areas. Of the 104 participants with diabetes, 87% reported MLTCs. Of the 146 men reporting ever having a doctor diagnosed mental health condition, 82 (56%) reported at least one other obesity-related condition.

**Table 2:** Coexistence of long-term conditions, disability and living in an area of deprivation

Participants (n=583)	Diabetes present (n=104)	Mental health condition present (n=146)	Disability present*(n=165)	Some impairment: yes to disability question 1 only (n=68)	Lives in the 2 more deprived quintiles for Index of Multiple Deprivation† (n=225)
No-long-term conditions (n=167)	–	–	12 (7.3)	8 (11.8)	65 (28.9)
One long-term condition (n=181)	14 (13.5)	64 (43.8)	54 (32.7)	26 (38.2)	67 (29.8)
Two or more long-term conditions (n=235)	90 (86.5)	82 (56.2)	99 (60.0)	34 (50.0)	93 (41.3)

* Office for National Statistics definition.

† Detail about analysis for Index of Multiple Deprivation is provided elsewhere.

A 12-month weight measurement (the primary outcome for the trial) was recorded for 73% of the 583 trial participants included (table 3). Of participants providing a weight measurement at 12 months, 125 (75%) reported no obesity-related long-term conditions, 130 (72%) a single condition, 170 (72%) MLTCs, 68 (65%) diabetes and 125 (76%) a disability. Of the 104 men with diabetes who enrolled in the trial, 23 (22%) men declined consent for providing a 12-month weight, double the proportion in the other long-term condition categories. The proportions retained across long-term condition categories were similar.

**Table 3:** Retention of participants with long-term conditions and disability at the 12-month weight assessment

12-month follow-up status, n (%)	No long-term conditions n=167	Single long-term conditions n=181	Multiple long-term conditions n=235	Diabetes n=104	Disability n=165	Trial participants n=583
Provided a 12-month weight	125 (75)	130 (72)	170 (72)	68 (65)	125 (76)	425 (73)
Attended weight verification face to face per protocol*	120 (72)	119 (66)	157 (67)	62 (60)	117 (71)	396 (68)
Lost to follow-up	24 (14)	34 (19)	36 (15)	13 (13)	23 (14)	94 (16)
Declined providing a weight at 12 months	18 (11)	17 (9)	29 (12)	23 (22)	17 (10)	64 (11)

* Per protocol entailed attending within 23 days of the 12-month weight loss target date with weight taken on study scales.

The secondary trial outcomes were quality of life (EuroQol 5-Dimension 5-Level (EQ-5D-5L)), Warwick-Edinburgh Mental Well-being Scale,^32 33^ Weight Self-Stigma Questionnaire^34^; for anxiety and depression the Patient Health Questionnaire-4 item (PHQ-4)^35^ and the anxiety and depression subscale of the EQ-5D-5L.^36^ Comparisons between the secondary outcomes for the text messages with incentives group compared with the control group, and between the text messages alone group and the control group, showed no difference.^19^ There was a 5-point improvement in health on the EQ-5D-5L 0–100 Visual Analogue Score (VAS) at 12 months for the text messages with financial incentives group compared with the control group. For men living with MLTCs there were small reductions in weight stigma across all trial groups and a small improvement in well-being and EQ-5D-5L VAS scores for the intervention groups (online supplemental file S4: Mental health and wellbeing at baseline and 12 months by participant long-term condition (LTC) status). When descriptively comparing men with multiple, single or no long-term conditions, the directions of change in mental health and well-being scores were inconsistent.

The 235 (40%) men with MLTCs in the trial had 153 medical conditions and reported 182 (50%) of the total 366 harms recorded,^19^ including 29 social harms. They reported 60 (45%) of the 132 unexpected benefits from participating in the trial. The harms and benefits database identified obesity-related conditions not specifically sought at baseline, including fatty liver disease, gallbladder disease, kidney stones and sleep apnoea, in addition to disabling long-term conditions where obesity is not an obvious risk factor, for example, Parkinson’s disease, respiratory conditions, visual impairment and bladder catheters.

### Qualitative findings

Of the 54 men who were interviewed, 27 reported MLTCs and 21 reported a disability (table 4). There was overlap between the reporting of MLTCs, mental health conditions and disability in the trial population and in the qualitative sample (figure 1). MLTCs often co-occurred with mental health conditions (figure 1), and some participants living with MLTCs, but no doctor diagnosed mental health condition, described periods of depression or low mood.

**Table 4:** Characteristics of participants (n=54) taking part in qualitative interviews at 12 months

Characteristic	Text messages with financial incentive	Text messages alone	Total
Less deprived (Index of Multiple Deprivation quintiles 3–5)	18	13	31
More deprived (Index of Multiple Deprivation quintiles 1 and 2)	12	10	22
Multiple long-term conditions present at baseline	17	10	27
Single long-term condition present at baseline	8	6	14
No long-term conditions present at baseline	5	8	13
Disability present at baseline	14	7	21
Weight trajectory—meets all or some 5% or 10% targets	21	7	28
Weight trajectory—meets no 5% or 10% targets	9	17	26

Within both intervention groups, participants with MLTCs had similar experiences of the interventions and managing their weight compared with those without MLTCs. The texts with financial incentives and the texts alone interventions appealed and helped some to lose weight, but not others. This finding is consistent with the results of the trial subgroup moderator analyses which showed no differential effectiveness for per cent weight loss at 12 months according to the presence or absence of single or MLTCs or disability.^31^ Key themes with illustrated interview quotations and participant reports relate to unexpected benefits and harms, barriers to weight loss, experiences of those who did not meet targets and accessibility.

Developing long-term conditions or adverse health events could be a pivotal time for men to join the trial or a motivator for behaviour change when participating.^37^ Fear of the future, accounts of premature morbidity in family members and a revelation during consultations with health professionals that obesity is linked to and possibly the cause of poor health were motivators for some.

My consultant had said to me that obviously you’ve got a [device fitted] and you need to take care and one of the things that you should do truthfully is maybe try and lose a bit of weight because that will always help. So that was why I embarked on that particular programme [Game of Stones]*.* (Interview 13014, texts alone, met targets)

Several men spoke about how taking part in Game of Stones had multiple unexpected benefits on their long-term conditions, health and well-being. The development of long-term conditions was an added motivator to continue their weight loss efforts, and one that was for some prioritised over and above the financial incentives. Several participants described sustained health benefits in terms of reversal of diabetes or pre-diabetes, reducing pain, diabetic or blood pressure medications, no longer requiring joint replacement surgery and accounts of health professional praise.

Can do shoe laces up and can get out of bath normally. Much fitter - walking better. Can balance on motorcycle better. Eyesight better - particularly night vision. Can wear normal shoes. Legs are normal. - much fewer diabetic ulcers. (Fieldworker notes, texts with incentives, met targets)

Although men with diabetes had lower retention in the trial, several engaged with the interventions and reported health and social benefits. Benefits could extend beyond the participant to family members by, for example, changing their eating behaviours, and men described how they were now able to pick up their children.

A new outlook and a new awareness he wouldn't have had without the programme. Him and his [spouse] have completely changed what they eat. (Fieldworker notes, texts alone, met some targets)

There were many examples of changes made to individual or family eating patterns, and a beneficial focus on weight loss goals, with some in the texts with incentive group reporting unexpected support from family and friends.

Encouragement from [sibling], [spouse] and [parent], wasn’t expecting so much support. [Sibling] rang his [parent] to tell [them] how well he was doing. Didn’t expect as much weight loss especially as the last 3 months have been difficult due to his [close family member] dying. Said he had re-found his willpower*.* (Fieldworker notes, texts with incentives, met targets)

However, several men spoke about MLTCs and their health as barriers to weight loss, regardless of whether they met their weight loss targets or not (box 1). Men spoke of medication for long-term conditions leading to weight gain or the requirement to take medication regularly with food as barriers to managing food intake. Periods of severe illness or hospitalisation represented a setback in weight loss efforts, with men feeling they had no control over food or exercise while unwell. New adverse events during the trial could change a weight trajectory, particularly when loss of mobility occurred:

Participant said it was unexpected that his diabetes nurse was so pleased with him, as pre-study they were talking about putting him on insulin due to blood sugar management. Now his HBA1C levels are nearly the same as a non-diabetic person. Can consider getting a tattoo now blood sugars are more stable as previously could not heal cuts. (Fieldworker notes, texts alone, met some targets)

Box 1 **Multiple long-term conditions and resulting poor health as barriers to losing weight**

- ‘Had it not been for the fact I was taking medication five times a day and I had to take them with something to eat made it hard to cut down on food when you're having to eat five times a day.’ (Interview 11184, texts with incentives, met no targets)
- ‘I think I just come out of surgery at that stage as well because I had a really bad experience during the year…so I was in hospital for about a week and I was just reading the texts and was like this really isn’t relevant because I've been eating hospital food and there's nothing I can do here …’ (Interview 13035, texts with incentives, met some targets, mental health condition with a non-obesity related long-term condition*)
- ‘Yeah, well I’ve got [a skin condition] and it affects my knees very badly. I often have [infections] and that can be very debilitating and doesn’t allow me to be able to walk very far. And I’ve got…breathing problems…I look at a set of stairs and I don’t want to go up them because it’s too painful… Or do I walk up the road with the dog, or do I think that actually don’t do that, that’s just gonna hurt? Whatever it might be, little choices that you make all the time that become more and more. You know the decision that you make is more don’t do it, don’t move, don’t stand up, don’t try to exert yourself because you’re just gonna be in more pain, so you know…’ (Interview 12078, texts alone, met no targets)
- ‘I have mobility issues, which gradually got worse. And again it got to the stage where I had to go for a…[joint] operation…I struggled then to walk. First of all I was on a stick, then I was on crutches. I was on a zimmer frame for a while. I just lost the crutches this week and hopefully…I’m moving… I do have memory problems ever since I got Covid, part of the Covid thing’ (Interview 13171, texts alone, met no targets).

*All other quotations are from men with obesity-associated multiple long-term conditions.

The text messages had a key message for men: although many would like to lose weight through exercise alone, research has shown that it has little effect in the absence of dietary change.^4^ The text messages emphasised the importance of exercise for maintaining weight already lost and for improving overall health and well-being. However, for many, their poor physical health was a barrier to exercise, and some felt exercising was key to weight loss. If physical activity was perceived as beyond their ability, they did not see the point of engaging with strategies related to changing food consumption.

It’s just difficult to lose weight when you can't move around as much as you'd like to……especially when your mobility certainly can't go out as much as you would like to and this kind of thing, one turns towards the fridge. (Interview 12181, texts alone, met no targets)

From the interview, fieldworker notes and harms and benefits data it was evident that some men were going through challenging times and living through difficult circumstances. Social harms included bereavement of family, friends and pets, stressful employment situations, unemployment, financial concerns and caring responsibilities. For some, combinations of MLTCs, disability and adverse social events clustered together creating competing priorities. These life events could become their focus and take precedence over trying to lose weight.

…there was so many things happened. My [close relative] had a serious health issue…and so much so that I had to in effect stop the divorce process because I had to focus…And then I had a few health issues, you know, and you know, both physical but also had [a serious health event requiring hospital admission] essentially…so it kind of knocks you a bit… (Interview 11226, texts with incentives, met no targets)

Either from accounts in interviews, or when reporting harms and unexpected events, problems with mobility because of arthritis, pain and breathing difficulties were common, alongside injuries and illnesses, such as COVID-19.

It’s just I didn't give it [Game of Stones] my full attention because I was going through a wee bit of depression during it, but it was still beneficial, so… we’ d just had the remnants of lock-down, I think it was. When I wasn’ t feeling depressed, I was sticking to the plan, I was eating healthy and all that; when I was depressed, I wasn't on it at all. I was like eating pizza, eating rubbish basically. (Interview 11086, texts with incentives, met some targets)

Men who did not meet weight targets reported some positive behaviour changes prompted by receiving text messages and many reported deriving some benefit. However, texts did not appeal to others, and some felt stressed trying to reach weight goals. Accessibility and acceptability for people with poor health, long-term conditions or disability was a consideration when designing Game of Stones.^18^ Trial retention and attendance at assessments was consistent with other weight management trials in primary care.^19 38^ Overall, interview accounts with men living with MLTCs and/or disability found the in-person weight assessments acceptable. Appointments were valued if they were easy to make, convenient, flexible and in familiar local venues, particularly because to gain the financial incentive, weight needed to be recorded using study scales within 3 weeks of the weight loss target date. Although stigma scores decreased in all three trial groups between baseline and 12 months, there were accounts within the interviews that indicated that men recognised obesity as a cause of ill health, and some believed that it was self-inflicted and people with obesity are to blame for additional burden on the NHS.

I mean if somebody has a heart attack because they're overweight and under exercised, that costs a fortune because you've got the whole medical team from A& E through to the surgeons, you know doing XYZ. And that could be argued as partly self-inflicted. (Interview 11200, texts with incentives, met targets)

PPI at the start of the trial resulted in a change to the trial protocol due to the COVID-19 pandemic. Men who did not have their own transport, and who were anxious about travelling on public transport due to the infection risk were offered a taxi service to attend a local venue for their weight assessments. A participant with diabetes and poor mobility, who had a daily carer to help him to live independently, provided feedback that this taxi service had been crucial to him continuing to engage in Game of Stones.

## Discussion

The Game of Stones trial recruited men with high levels of MLTC and disability. Compared with men with no long-term conditions, men with MLTCs and/or disability were older, fewer had a degree-level qualification, and fewer were in full-time work. There was considerable overlap for men reporting MLTCs, mental health conditions, disability and living in more disadvantaged areas. Overall trial retention was 73%, with more men with MLTCs (72%) and/or disability (76%) retained compared with men with diabetes (65%). In the moderator analyses for the primary outcome of percentage weight loss at 12 months, the 92 men with disabilities were noted to have done well, although the difference was small and unlikely to be of clinical relevance.^31^ Weight stigma, well-being and quality-of-life scores improved or stayed the same between baseline and 12 months for men with MLTCs in the intervention groups; however, the PHQ-4 and EQ-5D-5L anxiety and depression scores were inconsistent. Participant experiences indicated complex dynamic health, social and life situations where the Game of Stones intervention provided motivation for some to lose weight but not for others.

This trial addresses concerns about inequality-generating interventions,^39^ for example, where in-person weight management programmes suit people with time, resources and ability to travel. Findings from the implementation of the UK Diabetes Prevention Programme showed that recruitment and retention were challenging, particularly for equity and inclusion.^40^ Detailed socioeconomic, disability and long-term conditions characteristics are reported here, unlike many weight management trials for men.^20^ This is potentially important, particularly for participants on lower incomes, where complex health and social situations may adversely impact attentional focus and self-care decision making.^41 42^ The four in-person contacts needed to assess weight over the 12-month intervention period, compared with the 12 recommended for primary care weight management interventions in a recent systematic review,^38^ may reduce some barriers experienced by people with disabilities, MLTCs or transport issues and help the NHS to meet increasing demand for obesity management services. A taxi made a difference to appointment attendance for some men on low income, with MLTCs/disability or requiring care. Inclusivity for such men is important. Game of Stones fits with recent UK recommendations for new UK digital weight management delivery platforms.^43^

This mixed-methods exploratory process evaluation is unusual by providing insights into the experiences of men living with MLTCs and/or disability, who are under-represented in behavioural weight management trials. The under-reporting of harms in behavioural weight management trials^44 45^ is addressed in the report of the trial results,^19^ with participant accounts of harms and benefits integrated with trial qualitative and quantitative data. There are challenges in the consistency of definitions for MLTCs across all health and social care research, with some not including mental health conditions.^46^ The obesity-related conditions asked about in our baseline questionnaire were limited compared with the 21 major diseases that we could have included.^2^ There is an association between questionnaire length and perceived relevance with retention in trials.^47^ To meet our aim of recruiting underserved men living in more disadvantaged areas, and with guidance from the target population via PPI, we made trade-offs between questionnaire burden and the extent of information sought in the trial. The overlap between MLTCs, mental health conditions and disability is evident. The ‘met weight loss targets’ classification, which was a requirement for incentive payments, oversimplified men’s weight loss trajectories and requires a cautious interpretation. For example, a participant with less than 5% weight loss throughout is classified as met no weight loss targets, whereas a man who lost 6% of starting weight at 3 months, but then subsequently lost less than 5% would be classified as met some targets, as would a man with weight loss of 5% at 3 months, 8% at 6 months and 9% at 12 months.

Our finding that more men with diabetes withdrew from the trial weight assessments is an important consideration for implementation. Some men with diabetes engaged and benefited from the interventions. However, the text messages were not designed specifically for men with diabetes and did not appeal to some. Other potential reasons for men with diabetes declining follow-up are concurrent regular follow-up by health services and being prescribed drugs such as glucagon like peptide 1 (GLP-1) receptor agonists to control their diabetes, resulting in weight loss. Sensitivity analyses in the trial for missing data and removing participants taking weight loss drugs or meal replacements did not change the trial effectiveness for percentage weight loss at 12 months.^19^ Large cohort studies illustrate the challenges of preventing complex multimorbidity if a single disease approach is undertaken.^2^ Game of Stones has a citizen first, universal ethos ranging from primary through to tertiary disease prevention, but with no professional gatekeepers required to join and minimal exclusion criteria. It is unclear whether a different approach is required for men with diabetes in a rapidly changing policy landscape.

## Conclusion

The Game of Stones text messages with or without financial incentives are acceptable, low burden, low cost and have been shown to be effective. The intervention can address prevention and management of obesity for men with multimorbidity and disability, which may be a useful approach compared with targeting individual conditions. Policy makers would need to decide how and whether GLP-1 receptor agonist health service prescribing for weight loss or for diabetes could integrate with providing Game of Stones, given the substantial weight loss effects experienced with these drugs. There is an opportunity for new interventions that target men living with obesity who experience lengthy periods of immobility due to injury, illness or prolonged hospital stays. Perceptions that inability to exercise renders it not worthwhile making dietary changes to lose weight could be amenable to behaviour change and assist with weight loss maintenance.

## Supplementary material

10.1136/bmjopen-2025-113802 online supplemental file 1

## Data Availability

Data are available upon reasonable request.

## References

[1] Lingvay I, Cohen RV, Roux CW le (2024). Obesity in adults. Lancet.

[2] Kivimäki M, Strandberg T, Pentti J (2022). Body-mass index and risk of obesity-related complex multimorbidity: an observational multicohort study. Lancet Diabetes Endocrinol.

[3] Sattar N, McMurray JJV, McInnes IB (2023). Treating chronic diseases without tackling excess adiposity promotes multimorbidity. Lancet Diabetes Endocrinol.

[4] Robertson C, Archibald D, Avenell A (2014). Systematic reviews of and integrated report on the quantitative, qualitative and economic evidence base for the management of obesity in men. Health Technol Assess.

[5] Office for National Statistics (ONS) (2024). National life tables–life expectancy in the UK. https://www.ons.gov.uk/peoplepopulationandcommunity/birthsdeathsandmarriages/lifeexpectancies/bulletins/nationallifetablesunitedkingdom/2020to2022.

[6] Hiam L, Klaber B, Sowemimo A (2024). NHS and the whole of society must act on social determinants of health for a healthier future. BMJ.

[7] Marmot M (2020). Health equity in England: the Marmot review 10 years on. BMJ.

[8] O’Callaghan CA, Rayner JJ, Thanabalasingham G (2025). Integrating and defragmenting multi-specialty care for people with multiple long-term conditions. Br J Hosp Med (Lond).

[9] Holt S, Santer M, Everitt H (2024). Clustering by health and social care need in multiple long-term conditions (MLTC): an exploratory qualitative study to understand the views of professionals and people living with MLTC about future interventions. Br J Gen Pract.

[10] De Soyza A, Goodwin VA, Roberts K (2025). Rising to the challenges of research in multiple long-term conditions: health and social care research ecosystem approaches. BMJ Open.

[11] House of Commons Library (2024). UK disability statistics: prevalence and life experiences. https://commonslibrary.parliament.uk/research-briefings/cbp-9602/#:~:text=The%20prevalence%20of%20disability%20rises%20with%20age%3A%20in,people%20aged%2085%20or%20over%20reported%20a%20disability.

[12] Dombrowski SU, Avenell A, Sniehott FF (2010). Behavioural interventions for obese adults with additional risk factors for morbidity: systematic review of effects on behaviour, weight and disease risk factors. Obes Facts.

[13] Kraef C, van der Meirschen M, Free C (2020). Digital telemedicine interventions for patients with multimorbidity: a systematic review and meta-analysis. BMJ Open.

[14] Browne S, Minozzi S, Bellisario C (2019). Effectiveness of interventions aimed at improving dietary behaviours among people at higher risk of or with chronic non-communicable diseases: an overview of systematic reviews. Eur J Clin Nutr.

[15] Cooper L, Ryan CG, Ells LJ (2018). Weight loss interventions for adults with overweight/obesity and chronic musculoskeletal pain: a mixed methods systematic review. Obes Rev.

[16] Nowakowska M, Zghebi SS, Ashcroft DM (2019). The comorbidity burden of type 2 diabetes mellitus: patterns, clusters and predictions from a large English primary care cohort. BMC Med.

[17] Hunt K, Wyke S, Gray CM (2014). A gender-sensitised weight loss and healthy living programme for overweight and obese men delivered by Scottish Premier League football clubs (FFIT): a pragmatic randomised controlled trial. Lancet.

[18] Dombrowski SU, McDonald M, van der Pol M (2020). Text messaging and financial incentives to encourage weight loss in men with obesity: the Game of Stones feasibility RCT. Public Health Res.

[19] Hoddinott P, O’Dolan C, Macaulay L (2024). Text messages with financial incentives for men with obesity: a randomised clinical trial. JAMA.

[20] McDonald MD, Hunt K, Sivaramakrishnan H (2022). A systematic review examining socioeconomic factors in trials of interventions for men that report weight as an outcome. Obes Rev.

[21] Dombrowski SU, Hoddinott P, Macaulay L (2025). Secondary analysis of the Game of Stones trial for men with obesity: examining moderator effects and exploratory outcomes. Obesity (Silver Spring).

[22] Macaulay L, O’Dolan C, Avenell A (2022). Effectiveness and cost-effectiveness of text messages with or without endowment incentives for weight management in men with obesity (Game of Stones): study protocol for a randomised controlled trial. Trials.

[23] O’Cathain A, Murphy E, Nicholl J (2010). Three techniques for integrating data in mixed methods studies. BMJ.

[24] Skivington K, Matthews L, Simpson SA (2021). A new framework for developing and evaluating complex interventions: update of Medical Research Council guidance. BMJ.

[25] Office for National Statistics (ONS) (2024). Classifications and harmonisation. https://www.ons.gov.uk/methodology/classificationsandstandards.

[26] Torrens CE, Turner K, Swingler J (2024). Mental health and weight loss in men: an exploratory mixed methods study of the Game of Stones trial. Public Global Health.

[27] Junqueira DR, Zorzela L, Golder S (2023). CONSORT Harms 2022 statement, explanation, and elaboration: updated guideline for the reporting of harms in randomised trials. BMJ.

[28] Mackenzie RM, Ells LJ, Simpson SA (2020). Core outcome set for behavioural weight management interventions for adults with overweight and obesity: standardised reporting of lifestyle weight management interventions to aid evaluation (STAR-LITE). Obes Rev.

[29] Torrens C, Turner K, Swingler J Mental health and the Game of Stones text messaging and financial incentives trial for weight loss in men: an exploratory mixed methods study (unpublished).

[30] Gale NK, Heath G, Cameron E (2013). Using the framework method for the analysis of qualitative data in multi-disciplinary health research. BMC Med Res Methodol.

[31] Dombrowski S, Hoddinott P, Macaulay L Secondary analysis of the Game of Stones trial of text messages with financial incentives for men with obesity Obesity (under review). Obesity.

[32] Tennant R, Hiller L, Fishwick R (2007). The Warwick-Edinburgh Mental Well-being Scale (WEMWBS): development and UK validation. Health Qual Life Outcomes.

[33] Bianco D (2012). Performance of the Warwick-Edinburgh Mental Well-Being Scale (WEMWBS) as a screening tool for depression in UK and Italy Università Di Bologna.

[34] Lillis J, Luoma JB, Levin ME (2010). Measuring weight self-stigma: the weight self-stigma questionnaire. Obesity (Silver Spring).

[35] Löwe B, Wahl I, Rose M (2010). A 4-item measure of depression and anxiety: validation and standardisation of the Patient Health Questionnaire-4 (PHQ-4) in the general population. J Affect Disord.

[36] Herdman M, Gudex C, Lloyd A (2011). Development and preliminary testing of the new five-level version of EQ-5D (EQ-5D-5L). Qual Life Res.

[37] The i Paper (2024). I was paid £400 to lose weight by the NHS - and it worked.

[38] Madigan CD, Graham HE, Sturgiss E (2022). Effectiveness of weight management interventions for adults delivered in primary care: systematic review and meta-analysis of randomised controlled trials. BMJ.

[39] Lorenc T, Petticrew M, Welch V (2013). What types of interventions generate inequalities? Evidence from systematic reviews. J Epidemiol Community Health.

[40] Barron E, Clark R, Hewings R (2018). Progress of the Healthier You: NHS Diabetes Prevention Programme: referrals, uptake and participant characteristics. Diabet Med.

[41] Appelhans BM (2023). The Cognitive Burden of Poverty: a mechanism of socioeconomic health disparities. Am J Prev Med.

[42] de Bruijn E-J, Antonides G (2022). Poverty and economic decision making: a review of scarcity theory. Theory Decis.

[43] National Institute for Health and Care Excellence (NICE) (2024). Digital technologies for delivering multidisciplinary weight-management services: early value assessment. https://www.nice.org.uk/guidance/hte14.

[44] Papaioannou D, Cooper C, Mooney C (2021). Adverse event recording failed to reflect potential harms: a review of trial protocols of behavioural, lifestyle and psychological therapy interventions. J Clin Epidemiol.

[45] Papaioannou D, Hamer-Kiwacz S, Mooney C (2024). Recording harms in randomised controlled trials of behaviour change interventions: a scoping review and map of the evidence. J Clin Epidemiol.

[46] The Academy of Medical Sciences (2018). Multimorbidity: a priority for global health research. https://acmedsci.ac.uk.

[47] Brueton VC, Tierney JF, Stenning S (2014). Strategies to improve retention in randomised trials: a Cochrane systematic review and meta-analysis. BMJ Open.

