# Supplementary material for: Men’s experiences of multiple long-term conditions and/or disability in the UK Game of Stones weight management trial: a mixed-methods evaluation
Source: BMJ Open. 2026 Sep 15;16(9):e113802. doi: 10.1136/bmjopen-2025-113802 (PMC13583699; doi:10.1136/bmjopen-2025-113802)
Supplement: Supplementary file 2 [file bmjopen-2025-113802.reviewer_comments.pdf]

PEER REVIEW HISTORY

BMJ Open publishes all reviews undertaken for accepted manuscripts. Reviewers are asked to complete a checklist review form and are provided with free text boxes to elaborate on their assessment. These free text comments are reproduced below.

ARTICLE DETAILS

Title (Provisional)

Men's experiences of multiple long-term conditions and/or disability in the UK Game of Stones weight management trial: a mixed methods evaluation

Authors

Macaulay, Lisa; O'dolan, Catriona; Swingler, James; Torrens, Claire; MacLean, Alice; Turner, Katrina; Avenell, Alison; Cotton, Seonaidh; Dombrowski, Stephan U; Gray, Cindy; Hunt, Kate; Kee, Frank; McKinley, Michelle C.; MacLennan, Graeme; Hoddinott, Pat

---

VERSION 1 - REVIEW

**Reviewer**	**1**
**Name**	**Shahbazi, Fatemeh**
**Affiliation**	**Hamadan University of Medical Sciences, Department of Epidemiology**
**Date**	**29-Jun-2026**
**COI**	**None**

---

1. The title is vague. What is the means of "long-term conditions in the Game of Stones text Messaging" and "financial incentives trial"? if is it possible please rewrite it. You can also use references #17 and 18 of this article to rewrite the title.
2. What is the mean of "secondary mental health" at the abstract. Its vague. Please clarify it.
3. Please report mean ± SD of age for participants in the abstract results.
4. How assessed "weight stigma, wellbeing and quality of life scores" by self-report and how scoring them? Answer to this question should be added to the abstract method.
5. At introduction everything is good and clear until you write "Game of Stones trial"!. I don't understand this phrase please more clarify it.
6. Why do lines 37 to 50 of the introduction sound like the findings of the current study? These results were also presented exactly in the results section of the abstract? Please correct it or clarify it.
7. Although the authors state that information on mental health is provided elsewhere, the section that is relevant to the current study is ambiguous. Why is the sentence

"Has a doctor ever told you that you have…" incomplete in the method? What is the purpose of writing it in the method?

8. Why were participants given a list of obesity-related diseases? What was the purpose of this? The second paragraph of the method, if it is relevant to the current study, please rewrite it and explain it in more detail.
9. Please add the text of the SMS that was sent to the participants to the method.
10. Was the mental health condition of the patient measured? Although it was mentioned in another study, please briefly explain it in the method section of this study.
11. Is it necessary to mention that the study is a clinical trial? If the authors do not present their analysis by arm, please correct this.
12. The authors should explain how they selected 54 of the 583 obese men who formed the initial population for the qualitative part of the study.
13. What were the interview questions in the qualitative part? All of these questions should be added to the method with details.
14. What statistics were used in the quantitative section?
15. What software or approaches were used for analysis in the quantitative and qualitative sections?

**Reviewer**	**2**
**Name**	**Jones, Christopher**
**Affiliation**	**Brighton and Sussex Medical School, Medical Statistics**
**Date**	**06-Jul-2026**
**COI**	**None**

This manuscript presents qualitative and extended quantitative results from a trial published separately. I was confused initially as I don't think the title or abstract make this clear enough – I was expecting comparisons between arms, rather than descriptive results from different groupings. This makes sense in the context, but I had to read on to understand this was not presenting the main trial results - this should be immediately clear.

Overall I have no particular issues with the statistics or analyses and results presented. They are relatively niche as they are not the main trial results, but they are informative and not over-analysed. It is efficient use of data collected from a trial. The statistics used are appropriate, as descriptive statistics always are. The decision to not perform statistical testing here is sensible.

The only issue I'd raise is the use of "significance" language. Statistical testing was not performed in this manuscript, but results from the main trial are discussed in terms of "statistical significance". Emphasis should be on magnitude of the effect and weight of evidence against the null hypotheses (i.e. interpreting p-values on a continuous scale).

Minor points

Table 2, bottom right cell is missing an open parenthesis.

VERSION 1 - AUTHOR RESPONSE

Reviewer: 1

Comments to the Author:

1. The title is vague. What is the means of "long-term conditions in the Game of Stones text Messaging" and "financial incentives trial"? if is it possible please rewrite it. You can also use references #17 and 18 of this article to rewrite the title.

The title has been re-written to reflect the research question, study design and setting: 'Men's experiences of multiple long-term conditions and/or disability in the UK Game of Stones weight management trial: a mixed methods evaluation'

2. What is the mean of "secondary mental health" at the abstract. Its vague. Please clarify it.

This has been deleted for clarity as it was not critical for the abstract. Secondary outcomes in the Game of Stones trial included mental health but it is not the focus of this paper.

3. Please report mean ± SD of age for participants in the abstract results.

This has now been included in the abstract: '(mean [SD] age, 50.7 [13.3] years)'.

4. How assessed "weight stigma, wellbeing and quality of life scores" by self-report and how scoring them? Answer to this question should be added to the abstract method.

These measures were included in the baseline questionnaire of the main trial and have now been added to the abstract methods.

5. At introduction everything is good and clear until you write "Game of Stones trial"!. I don't understand this phrase please more clarify it.

This is the name of the main weight management trial on which the process evaluation was conducted. This has now been added to the title and abstract for clarity and the following added to the manuscript under Introduction: 'The Game of Stones weight management trial (hereafter referred to as the trial)'.

6. Why do lines 37 to 50 of the introduction sound like the findings of the current study?

These results were also presented exactly in the results section of the abstract? Please correct it or clarify it.

These are the results of the Game of Stones trial, which I have clarified in the text: 'The results of the trial showed that compared to the control group, men in the text messages with financial incentives group had a significantly larger mean percent weight loss…'. Thank you for pointing out the duplication in the abstract, this has now been rectified.

7. Although the authors state that information on mental health is provided elsewhere, the section that is relevant to the current study is ambiguous. Why is the sentence "Has a doctor ever told you that you have…" incomplete in the method? What is the purpose of writing it in the method?

The sentence including 'has a doctor ever told you' -has now been rephrased for clarity (page 4) and now reads: 'To establish participant long-term condition and disability status at baseline men were asked in the participant questionnaire to confirm if a doctor had ever told them that they have any of the following obesity related conditions by ticking a box: stroke/mini-stroke; high blood pressure; heart condition e.g. angina, atrial fibrillation; diabetes; cancer; arthritis; or a mental health condition.'

It was intended to highlight that the presence of obesity-related conditions was doctor-diagnosed and explain how MLTCs (multiple long-term conditions) were defined in this study.

8. Why were participants given a list of obesity-related diseases? What was the purpose of this? The second paragraph of the method, if it is relevant to the current study, please rewrite it and explain it in more detail.

The baseline questionnaire in the main trial gathered data on doctor-diagnosed obesity-related conditions from the participant and this was used to establish whether they had MLTCs. The methods of the Game of Stones trial are summarised on page 4, of which this process evaluation paper was a part. As stated in the first paragraph of the methods (page 4), the key methods in the main trial relevant to this paper have been summarised and more detail can be found in the Supporting Information (S1). The name of the trial has also been added to the title and introduction for clarity.

9. Please add the text of the SMS that was sent to the participants to the method.

If this refers to the daily behavioural text messages sent to the participants during the main trial, a sentence has been added to the introduction (page 3) to describe the text messages and refer to a published paper providing examples:

‘The text message design incorporated behaviour change strategies to predominantly support motivation, self-regulation, and long-term maintenance of behaviour change using a person-centred approach’.

‘Examples of the text messages and a full description of the trial findings are presented elsewhere’ (reference given).

10. Was the mental health condition of the patient measured? Although it was mentioned in another study, please briefly explain it in the method section of this study.

Presence of a mental health condition (diagnosed by a doctor) was included in the baseline questionnaire of the Game of Stones trial and contributed to the definition of ‘living with an MLTC’ if it was present along with one or more other conditions. More detail has been added to the methods section (page 4):

‘The exploratory process evaluation findings relating specifically to participants with a mental health condition (n=146, 25%) are reported elsewhere but concluded that mental health difficulties were experienced in different ways by men during the trial, were unique to the individual and were a barrier to behaviour change for some but not for others’ (reference to Torrens et al 2024, pre-print paper).

11. Is it necessary to mention that the study is a clinical trial? If the authors do not present their analysis by arm, please correct this.

Game of Stones was a clinical trial, but the term ‘clinical’ has been removed from the introduction to avoid confusion as this was only used once.

12. The authors should explain how they selected 54 of the 583 obese men who formed the initial population for the qualitative part of the study.

The following has been added to the methods section to describe the sampling for the interviews: ‘Men were purposively selected to ensure a diverse sample representative of each trial centre, intervention group, differing deprivation status in addition to those self-reporting long-term conditions’.

13. What were the interview questions in the qualitative part? All of these questions should be added to the method with details.

The interview questions and prompts are included in the Supplementary Information (S2) and the following text was added to the methods section on page 5: ‘A topic guide of questions and prompts was used, for example: has anything made it difficult for you

to take part in Game of Stones, and what did you think of being offered money for reaching weight loss targets? (S2: Game of Stones 12 Month Interview Topic Guide).'

14. What statistics were used in the quantitative section?

In this paper, only descriptive statistics were used for the quantitative data analysis. Where relevant, the findings of the main trial are reported (with reference to the main trial and protocol papers for statistical analysis).

15. What software or approaches were used for analysis in the quantitative and qualitative sections?

Both the quantitative (descriptive statistics) and qualitative analysis software (QSR NVivo v20) and approaches (Framework approach) used to analyse the data are described in the final two paragraphs of the methods section (pages 5-6).

Reviewer 2

Comments to the Author:
This manuscript presents qualitative and extended quantitative results from a trial published separately. I was confused initially as I don't think the title or abstract make this clear enough – I was expecting comparisons between arms, rather than descriptive results from different groupings. This makes sense in the context, but I had to read on to understand this was not presenting the main trial results - this should be immediately clear.

Amendments have been made to the title and abstract to clarify the difference between this process evaluation paper and the Game of Stones trial on which it is based. The new title is: 'Men's experiences of multiple long-term conditions and/or disability in the UK Game of Stones weight management trial: a mixed methods evaluation'. Please also see response to Reviewer 1, comments 1 and 5).

Overall I have no particular issues with the statistics or analyses and results presented. They are relatively niche as they are not the main trial results, but they are informative and not over-analysed. It is efficient use of data collected from a trial. The statistics used are appropriate, as descriptive statistics always are. The decision to not perform statistical testing here is sensible.

The only issue I'd raise is the use of "significance" language. Statistical testing was not performed in this manuscript, but results from the main trial are discussed in terms of

"statistical significance". Emphasis should be on magnitude of the effect and weight of evidence against the null hypotheses (i.e. interpreting p-values on a continuous scale).

Thank you for raising this comment. We agree that the use of significance needs to be carefully managed in reporting of results so as not to mislead the reader's overall interpretation of the findings. We do believe that is it important to include and note the significance of the main trial results in the introduction and since the results are referenced it does not give the reader reason to believe that statistical testing was conducted in this manuscript. We have removed the significance wording on page 10 to move focus towards the magnitude of the difference only. In the discussion on page 15 we have amended the wording to describe the difference as small and unlikely to be of clinical relevance.

Minor points
Table 2, bottom right cell is missing an open parenthesis.

Thank you, this has been rectified.

VERSION 2 - REVIEW

**Reviewer**	**1**
**Name**	**Shahbazi, Fatemeh**
**Affiliation**	**Hamadan University of Medical Sciences, Department of Epidemiology**
**Date**	**14-Aug-2026**
**COI**	

The authors revised all comments.
